# Impact of co-morbidity on reoperation or death within 90 days of surgery for oesophageal cancer

**DOI:** 10.1093/bjsopen/zraa035

**Published:** 2021-01-09

**Authors:** Z Cheng, A Johar, E Gottlieb-Vedi, M Nilsson, J Lagergren, P Lagergren

**Affiliations:** Surgical Care Science, Department of Molecular Medicine and Surgery, Karolinska Institutet, Karolinska University Hospital, Stockholm, Sweden; Surgical Care Science, Department of Molecular Medicine and Surgery, Karolinska Institutet, Karolinska University Hospital, Stockholm, Sweden; Upper Gastrointestinal Surgery, Department of Molecular Medicine and Surgery, Karolinska Institutet, Karolinska University Hospital, Stockholm, Sweden; Division of Surgery, Department of Clinical Science, Intervention and Technology (CLINTEC), Karolinska Institutet, Stockholm, Sweden; Department of Upper Abdominal Diseases, Karolinska University Hospital, Stockholm, Sweden; Upper Gastrointestinal Surgery, Department of Molecular Medicine and Surgery, Karolinska Institutet, Karolinska University Hospital, Stockholm, Sweden; School of Cancer and Pharmaceutical Sciences, King's College London, London, UK; Surgical Care Science, Department of Molecular Medicine and Surgery, Karolinska Institutet, Karolinska University Hospital, Stockholm, Sweden; Department of Surgery and Cancer, Imperial College London, London, UK

## Abstract

**Background:**

The impact of preoperative co-morbidity on postoperative outcomes in patients with oesophageal cancer is uncertain. A population-based and nationwide cohort study was conducted to assess the influence of preoperative co-morbidity on the risk of reoperation or mortality within 90 days of surgery for oesophageal cancer.

**Methods:**

This study enrolled 98 per cent of patients who had oesophageal cancer surgery between 1987 and 2015 in Sweden. Modified Poisson regression models provided risk ratios (RRs) with 95 per cent confidence intervals (c.i.) to estimate associations between co-morbidity and risk of reoperation or death within 90 days of oesophagectomy. The RRs were adjusted for age, sex, educational level, pathological tumour stage, neoadjuvant therapy, annual hospital volume, tumour histology and calendar period of surgery.

**Results:**

Among 2576 patients, 446 (17.3 per cent) underwent reoperation or died within 90 days of oesophagectomy. Patients with a Charlson Co-morbidity Index (CCI) score of 2 or more had an increased risk of reoperation or death compared with those with a CCI score of 0 (RR 1.78, 95 per cent c.i. 1.44 to 2.20), and the risk increased on average by 27 per cent for each point increase of the CCI (RR 1.27, 1.18 to 1.37). The RR was increased in patients with pulmonary disease (RR 1.66, 1.36 to 2.04), cardiac disease (RR 1.37, 1.08 to 1.73), diabetes (RR 1.50, 1.14 to 1.99) and cerebral disease (RR 1.40, 1.06 to 1.85).

**Conclusion:**

Co-morbidity in general, and pulmonary disease, cardiac disease, diabetes and cerebral disease in particular, increased the risk of reoperation or death within 90 days of oesophageal cancer surgery. This highlights the value of tailored patient selection, preoperative preparation and postoperative care.

## Teaser

The impact of preoperative co-morbidity on postoperative outcomes in patients with oesophageal cancer is uncertain. A population-based and nationwide cohort study enrolled 98 per cent of all patients who had oesophageal cancer surgery in 1987–2015 in Sweden was conducted. Results showed that co-morbidity in general, and pulmonary disease, cardiac disease, diabetes and cerebral disease in particular, increased the risk of reoperation or death within 90 days of oesophageal cancer surgery. This highlights the value of tailored patient selection, preoperative preparation and postoperative follow-up.

## Resumen

**Antecedentes:**

El impacto de la comorbilidad preoperatoria en los resultados postoperatorios de pacientes con cáncer de esófago es incierto. Se llevó a cabo un estudio de cohortes de base poblacional y nacional para evaluar la influencia de la comorbilidad preoperatoria en el riesgo de reoperación o mortalidad durante los primeros 90 días tras cirugía por cáncer de esófago.

**Métodos:**

Este estudio reclutó 98% de los pacientes sometidos a cirugía del cáncer de esófago entre 1987 y 2015 en Suecia. Los modelos de regresión de Poisson modificados proporcionaron las tasas de riesgo (*risk ratios*, RRs) con los i.c. del 95% para estimar las asociaciones entre comorbilidad y riesgo de reoperación o muerte a los 90 días de la esofaguectomía. Los RRs se ajustaron por edad, sexo, nivel de educación, estadio patológico del tumor, tratamiento neoadyuvante, volumen anual del hospital, histología del tumor y periodo en el que se realizó la cirugía.

**Resultados:**

De un total de 2.576 pacientes, 446 (17,3%) precisaron reoperación o fallecieron durante los primeros 90 días tras la esofaguectomía. Los pacientes con un índice de comorbilidad de Charlson ≥ 2 presentaron un riesgo aumentado de reoperación o muerte en comparación con los pacientes con índice de comorbilidad de 0 (RR = 1,78; i.c. del 95%: 1,44-2,20), y el riesgo aumentó en una media de un 27% por cada punto de incremento en el índice de comorbilidad (RR = 1,27; i.c. del 95%: 1,18-1,37). Los RRs aumentaron en pacientes con enfermedad pulmonar (RR =1,66; i.c. del 95%: 1,36-2,04), enfermedad cardiaca (RR = 1,37; i.c. del 95%: 1,08-1,73), diabetes (RR = 1,50; i.c. del 95%: 1,14-1,99) y enfermedad cerebral (RR = 1,40; i.c. del 95%: 1,06-1,85).

**Conclusión:**

La comorbilidad en general y, en particular, la enfermedad pulmonar, enfermedad cardiaca, diabetes y enfermedad cerebral aumentaron el riesgo de reoperación o muerte durante los primeros 90 días tras la cirugía del cáncer de esófago. Estos hallazgos ponen de relieve el valor de la selección individualizada de los pacientes, la preparación preoperatoria y los cuidados postoperatorios.

## Introduction

Oesophagectomy and neoadjuvant therapy is standard treatment for locally advanced oesophageal cancer without distant metastases^1^. Co-morbidity is present in around 40–60 per cent of patients who have an oesophagectomy^2^^,^^3^, and 40–50 per cent experience postoperative complications^4^^,^^5^.

Better knowledge about how co-morbidity influences outcome after oesophagectomy could help tailor clinical decision-making and modify preoperative preparation and postoperative care. Unlike variations in definition and different degrees of severity that exist for many of the complications that follow oesophagectomy, reoperation and short-term mortality are objective and accurately defined outcomes. When assessing short-term outcomes of oesophagectomy, most studies historically focused on in hospital or 30-day outcomes, although 90 days is nowadays often considered a better cut-off as a result of improvements in postoperative care^6^^,^^7^. Although co-morbidities are reported to increase the risk of 30-day complications in general, their influence on outcome at 90 days is less clear, and even less information has been reported on the influence of specific conditions, including diabetes, pulmonary disease, cerebral disease and cardiac disease^3^^,^^8–11^.

This study aimed to clarify how co-morbidity influences the risk of reoperation or mortality within 90 days of surgery for oesophageal cancer.

## Methods

This was a nationwide population-based cohort study of patients with oesophageal cancer who had an oesophagectomy in Sweden between 1 January 1987 and 31 December 2015. The study was approved by the Ethical Review Board in Stockholm, Sweden.

### Data source and data collection

Patients with oesophageal adenocarcinoma or squamous cell carcinoma were identified from the Swedish Cancer Registry, which includes at least 98 per cent of all patients with oesophageal cancer in Sweden^12^. To select the patients who had undergone oesophagectomy, the database was linked with the national Swedish Patient Registry, which has a 99.6 per cent positive predictive value for oesophagectomy records^13^. Data on co-morbidity were also collected from the Swedish Patient Registry. Information on mortality was retrieved from the Swedish Cause of Death Register with 100 per cent completeness^14^. Information regarding calendar period, hospital volume, neoadjuvant treatment, and tumour characteristics (pathological tumour stage and tumour histology) was collected by review of all histopathology reports and operation charts according to a predefined protocol. Information about educational level was retrieved from the longitudinal integrated database for health insurance and labour market studies (LISA). Linkages of participants between registries and identification of their medical records were enabled by the individual unique Swedish personal identity number, a 10-digit number assigned to each Swedish resident^15^.

The study exposure was co-morbidity recorded before the date of the oesophagectomy, defined according to the most recent Charlson Co-morbidity Index (CCI)^16^^,^^17^. This validated index includes chronic, coexisting, and mainly non-communicable diseases, which are recorded using the diagnosis code according to the ICD classification. The following 14 co-morbidities were included: myocardial infarction, congestive heart failure, peripheral vascular disease, cerebrovascular disease, dementia, chronic pulmonary disease, rheumatic disease, liver disease, diabetes, hemiplegia/paraplegia, renal disease, malignancy, metastatic tumours, and acquired immune deficiency syndrome (AIDS). Oesophageal cancer diagnosis was excluded when calculating the CCI score.

The outcome was reoperation or all-cause death within 90 days of the oesophagectomy. This composite outcome was used to avoid competing risk of death when assessing reoperations, as any death within 90 days of surgery made it impossible to have a reoperation^18^.

The following eight co-variables were considered as potential confounders because they could influence both the exposure (co-morbidity) and the outcome (reoperation or death): age, sex (male or female), educational level (less than 9, 9–12 or more than 12 years of formal education), pathological tumour stage (0–I, II, III or IV in the 7th version of cancer staging manual by the American Joint Committee on Cancer), neoadjuvant therapy (no or yes), annual hospital volume (fewer than 10 or 10 or more operations per year), tumour histology (adenocarcinoma or squamous cell carcinoma), and calendar period of oesophagectomy.

### Statistical analysis

Co-morbidity was analysed in three ways: CCI score analysed as three separate categories (0, 1, or 2 or above); CCI score analysed as a discrete variable to explore linear trends; and five co-morbidity groups included in the CCI analysed separately (no or yes). The separate co-morbidity groups were: pulmonary disease (chronic bronchitis, emphysema, chronic obstructive pulmonary disorder, asthma, bronchiectasis, pneumoconiosis and chronic lung manifestations caused by chemicals, gases, smoke or radiation); cardiac disease (myocardial infarction and congestive heart failure); diabetes; cerebral disease (cerebrovascular disease, dementia, hemiplegia/paraplegia); and other malignancy (malignant lymphoma, leukaemia and solid malignant tumours, excluding oesophageal cancer and non-melanoma skin cancer). The reference category was patients without the specific co-morbidity.

A modified Poisson regression with robust error variance was used to calculate risk ratios (RRs) with 95 per cent confidence intervals (c.i.)^19^, adjusted for the co-variables listed and categorized as described above. Analyses stratified by median age (66 years or less and more than 66 years), annual hospital volume (fewer than 10 and 10 or more operations per year), tumour histology (adenocarcinoma and squamous cell carcinoma), and calendar period of surgery (1987–1999 and 2000–2015) were also conducted. In the stratified analyses, the RRs were adjusted for all eight co-variables, except for the stratification factor. When analysing the specific group of co-morbidities, further adjustment was made for other co-morbidities (no or yes), defined by the existence of other co-morbidities except for the analysed group. Interactions between pulmonary and cardiac disease were also explored in separate models. Because rates of missing data were low, complete case analyses were carried out. Two-sided tests at the 5 per cent level of significance were used for statistical testing. An experienced biostatistician was responsible for the statistical analyses, and SAS^®^ 9.4 (SAS Institute, Cary, NC, USA) software was used for all analyses.

## Results

The cohort included 2576 patients who had undergone oesophageal cancer surgery. Of these, 1553 patients (60.3 per cent) had at least one co-morbidity included in the CCI. The most common co-morbidity group was other malignancy (561 patients, 21.8 per cent), followed by pulmonary disease (386, 15.0 per cent), cardiac disease (308, 12.0 per cent), diabetes (238, 9.2 per cent), and cerebral disease (192, 7.5 per cent). Most co-variables were distributed evenly between patients with and those without co-morbidity (*Table 1*). In total, 446 patients (17.3 per cent) underwent reoperation or died within 90 days of the oesophagectomy: 195 (7.6 per cent) had a reoperation, 184 (7.1 per cent) died without reoperation, and 67 (2.6 per cent) underwent reoperation and died.

**Table 1:** Characteristics of 2576 study patients who underwent esophagectomy for oesophageal cancer in Sweden in 1987-2015

	Charlson Co-morbidity Index score		
	0 (*n*=1023)	1 (*n*=922)	≥ 2 (*n*=631)
Age (years)*	63.8(9.9)	65.7(9.1)	67.1(8.9)
**Sex**			
M	791 (77.3)	691 (74.9)	494 (78.3)
F	232 (22.7)	231 (25.1)	137 (21.7)
**Educational level (years)**			
<9	433 (42.3)	413 (44.8)	289 (45.8)
9–12	398 (38.9)	335 (36.3)	247 (39.1)
>12	156 (15.2)	145 (15.7)	83 (13.2)
Missing	36 (3.5)	29 (3.1)	12 (1.9)
**Pathological tumour stage**			
0–I	234 (22.9)	185 (20.1)	143 (22.7)
II	316 (30.9)	303 (32.9)	216 (34.2)
III	339 (33.1)	316 (34.3)	200 (31.7)
IV	63 (6.2)	59 (6.4)	42 (6.7)
Missing	71 (6.9)	59 (6.4)	30 (4.8)
**Neoadjuvant therapy**			
No	585 (57.2)	496 (53.8)	392 (62.1)
Yes	438 (42.8)	426 (46.2)	237 (37.6)
Missing	0 (0.0)	0 (0.0)	2 (0.3)
**Annual hospital volume**			
<10	446 (43.6)	393 (42.6)	269 (42.6)
≥10	577 (56.4)	529 (57.4)	362 (57.4)
**Tumour histology**			
Adenocarcinoma	575 (56.2)	462 (50.1)	356 (56.4)
Squamous cell carcinoma	444 (43.4)	458 (49.7)	272 (43.1)
Missing	4 (0.4)	2 (0.2)	3 (0.5)
**Calendar period**			
1987–1996	231 (22.6)	329 (35.7)	193 (30.6)
1997–2006	341 (33.3)	231 (25.1)	165 (26.1)
2007–2015	451 (44.1)	362 (39.3)	273 (43.3)

Values in parentheses are percentages unless indicated otherwise;

*values are mean(s.d.).

### Co-morbidity and risk of reoperation or death

Compared with patients with a CCI score 0, those with CCI score 2 or above had a 78 per cent increased risk of reoperation or death within 90 days of surgery (RR 1.78, 95 per cent c.i. 1.44 to 2.20) (*Table 2*). The risk increased by an average of 27 per cent for each additional CCI point (RR 1.27, 1.18 to 1.37).

**Table 2:** Co-morbidity and risk ratios for reoperation or death within 90 days of oesophageal cancer surgery

	Reoperation or death		**Crude** RR*	**Adjusted** RR*^†^
	No (*n*=2130)	Yes (*n*=446)		
**CCI score**				
0	885 (86.5)	138 (13.5)	1.00 (reference)	1.00 (reference)
1	774 (83.9)	148 (16.1)	1.19 (0.96, 1.47)	1.04 (0.83, 1.30)
≥2	471 (74.6)	160 (25.4)	1.88 (1.53, 2.31)	1.78 (1.44, 2.20)
**CCI^‡^**	–	–	1.28 (1.20, 1.38)	1.27 (1.18, 1.37)
**Co-morbidity group**				
Pulmonary disease				
No	1841 (84.1)	349 (15.9)	1.00 (reference)	1.00 (reference)
Yes	289 (74.9)	97 (25.1)	1.58 (1.29, 1.92)	1.66 (1.36, 2.04)
Cardiac disease				
No	1889 (83.3)	379 (16.7)	1.00 (reference)	1.00 (reference)
Yes	241 (78.2)	67 (21.8)	1.30 (1.03, 1.64)	1.37 (1.08, 1.73)
Diabetes				
No	1941 (83.0)	397 (17.0)	1.00 (reference)	1.00 (reference)
Yes	189 (79.4)	49 (20.6)	1.21 (0.93, 1.58)	1.50 (1.14, 1.99)
Cerebral disease				
No	1984 (83.2)	400 (16.8)	1.00 (reference)	1.00 (reference)
Yes	146 (76.0)	46 (24.0)	1.43 (1.09, 1.87)	1.40 (1.06, 1.85)
Other malignancy				
No	1673 (83.0)	342 (17.0)	1.00 (reference)	1.00 (reference)
Yes	457 (81.5)	104 (18.5)	1.09 (0.90, 1.33)	1.19 (0.97, 1.47)

Values in parentheses are percentages (reported across the row in each group) unless indicated otherwise; *values in parentheses are 95 per cent confidence intervals.

^†^Adjusted for age, sex, educational level, pathological tumour stage, neoadjuvant therapy, annual hospital volume, tumour histology and calendar period.

^‡^Analysed as a discrete variable to evaluate the linear trend. RR, risk ratio.

Regarding the five specific co-morbidity groups, the RR of reoperation or death was increased among patients with pulmonary disease (RR 1.66, 95 per cent c.i. 1.36 to 2.04), cardiac disease (RR 1.37, 1.08 to 1.73), diabetes (RR 1.50, 1.14 to 1.99) and cerebral disease (RR 1.40, 1.06 to 1.85), but not among patients with other malignancy (*Table 2*).

The risks of reoperation or death among patients with higher CCI scores did not change much in the stratified analyses (*Table 3*). For specific co-morbidity groups, the risk estimates were particularly increased among patients with pulmonary disease who had oesophagectomy between 1987 and 1999 (RR 1.83, 95 per cent c.i. 1.40 to 2.39), and in patients with squamous cell carcinoma and diabetes (RR 1.85, 1.24 to 2.75) (*Table 4*). Analyses further adjusting for other co-morbidities showed similar estimates as those in *Tables 2–4*, and no statistically significant interactions between pulmonary and cardiac disease were found in the main or subgroup analyses (data not shown).

**Table 3:** Charlson Co-morbidity Index and risk ratios for reoperation or death within 90 days of oesophageal cancer surgery in stratified analyses

	No. of patients (*n*=2576)*	CCI score			CCI§
		0	1	≥ 2	
		Reference	RR^†‡^	RR^†‡^	RR^†‡^
**Age (years)**					
≤66	1189 (46.2)	1.00	1.02 (0.74, 1.40)	1.80 (1.34, 2.44)	1.27 (1.14, 1.41)
>66	1152 (44.7)	1.00	1.09 (0.79, 1.50)	1.81 (1.34, 2.43)	1.28 (1.16, 1.41)
**Annual hospital volume**					
<10	1043 (40.5)	1.00	1.11 (0.82, 1.49)	1.69 (1.27, 2.25)	1.26 (1.14, 1.40)
≥10	1298 (50.4)	1.00	0.98 (0.69, 1.38)	1.94 (1.41, 2.67)	1.30 (1.16, 1.45)
**Tumour histology**					
Adenocarcinoma	1271 (49.3)	1.00	0.81 (0.56, 1.17)	1.83 (1.34, 2.50)	1.31 (1.18, 1.46)
Squamous cell carcinoma	1070 (41.5)	1.00	1.22 (0.91, 1.62)	1.66 (1.24, 2.22)	1.21 (1.09, 1.34)
**Calendar period**					
1987–1999	924 (35.9)	1.00	1.08 (0.81, 1.44)	1.66 (1.25, 2.21)	1.23 (1.12, 1.36)
2000–2015	1417 (55.0)	1.00	1.05 (0.74, 1.48)	1.97 (1.43, 2.70)	1.32 (1.18, 1.46)

Values in parentheses are

*percentages (reported across the column in each stratified group; may not add to 100 per cent because of missing data) and ^†^95 per cent confidence intervals.

^‡^Adjusted for age, sex, educational level, pathological tumour stage, neoadjuvant therapy, annual hospital volume, tumour histology and calendar period, except the stratification variable in each model.

^§^Analysed as a discrete variable to evaluate the linear trend. RR, risks ratio.

**Table 4:** Co-morbidity groups and risk ratios for reoperation or death within 90 days of oesophageal cancer surgery in stratified analyses

	Risk ratio*					
	Pulmonary disease	Cardiac disease	Diabetes	Cerebral disease	Other malignancy	
**Age (years)**						
≤66	1.57 (1.15, 2.15)	1.30 (0.87, 1.95)	1.67 (1.12, 2.48)	1.49 (0.93, 2.38)	1.30 (0.96, 1.74)	
>66	1.79 (1.38, 2.32)	1.46 (1.10, 1.95)	1.44 (0.97, 2.12)	1.34 (0.95, 1.89)	1.09 (0.81, 1.46)	
**Annual hospital volume**					
<10	1.72 (1.33, 2.23)	1.32 (0.96, 1.82)	1.54 (1.04, 2.26)	1.45 (1.01, 2.07)	1.07 (0.79, 1.45)	
≥10	1.63 (1.18, 2.25)	1.46 (1.04, 2.06)	1.54 (1.03, 2.31)	1.35 (0.86, 2.11)	1.31 (0.97, 1.77)	
**Tumour histology**						
Adenocarcinoma	1.67 (1.23, 2.26)	1.42 (1.02, 1.99)	1.37 (0.95, 1.98)	1.53 (1.04, 2.26)	1.49 (1.12, 2.00)	
Squamous cell carcinoma	1.61 (1.23, 2.12)	1.26 (0.90, 1.76)	1.85 (1.24, 2.75)	1.27 (0.85, 1.89)	0.92 (0.68, 1.24)	
**Calendar period**						
1987–1999	1.83 (1.40, 2.39)	1.36 (0.98, 1.90)	1.56 (1.02, 2.40)	1.25 (0.83, 1.87)	0.96 (0.69, 1.34)	
2000–2015	1.46 (1.07, 1.99)	1.39 (0.99, 1.94)	1.53 (1.06, 2.19)	1.62 (1.11, 2.38)	1.39 (1.05, 1.84)	

Numbers of patients are as shown in *Table 3*. Values in parentheses are 95 per cent confidence intervals.

*Adjusted for age, sex, educational level, pathological tumour stage, neoadjuvant therapy, annual hospital volume, tumour histology and calendar period, except the stratification variable in each model.

## Discussion

This study has indicated that preoperative co-morbidity, measured as higher CCI scores, was associated with an increased risk of reoperation or death within 90 days of oesophageal cancer surgery. Of the specific co-morbidity groups, pulmonary disease, cardiac disease, diabetes and cerebral disease, but not other malignancy, were associated with worse 90-day outcomes.

Among the strengths of this study are the nationwide and population-based cohort design with complete inclusion and follow-up, along with detailed and high-quality data on exposures, co-variables and outcome. The outcome was assessed objectively, using a combination of reoperation and death to represent severe postoperative adverse outcomes, as well as to handle the competing risk of death on the incidence of reoperation. Among weaknesses is possible unmeasured or residual confounding in this observational study, although the main risk factors for poor short-term outcomes were controlled for in the analyses. Life-threatening complications requiring intensive care were not included in the outcome due to incomplete data records, but this could only dilute the effects and would not change the conclusions. Missing data were limited and evenly distributed, alleviating concerns about this influencing results. Although the CCI is widely accepted, it was not possible to assess the severity of co-morbidities in detail, or to examine co-morbidities not included in this index. Despite the large sample size, few patients selected for oesophagectomy had a high CCI score, which reduced the range of exposure and the statistical power in some subgroup analyses.

Most studies assessing co-morbidity in relation to short-term complications after oesophageal cancer surgery have focused on 30-day outcomes, and studies describing 90-day outcomes are sparse. Two Swedish studies and one from the USA found a preoperative CCI score of 2 or above to be associated with increased mortality or severe complications within 30 days of surgery^3^^,^^8^^,^^20^, in line with the present 90-day results. Regarding specific types of co-morbidity, a French cohort study^10^ of 3009 patients reported an increased risk of 30-day postoperative mortality among those with cardiovascular, but not pulmonary disease, and a recent European multicentre study^21^ of 1590 patients found that cardiorespiratory co-morbidity was associated with an increased risk of 30-day postoperative complications. In the present study, the finding that patients with cardiac or pulmonary disease had a higher rate of reoperation or death within 90 days of surgery may be due to the fact that patients with these co-morbidities have a relatively low performance status and are more susceptible to postoperative cardiorespiratory complications, such as arrhythmia and pneumonia, that account for about half of the in-hospital mortality after oesophagectomy^4^^,^^22^^,^^23^.

Studies have provided contradictory results regarding the impact of diabetes. A single-centre cohort study^11^ of 1282 patients from the Netherlands found no association between diabetes and 90-day mortality after oesophagectomy, which might be explained by well controlled perioperative glucose levels in a high-volume hospital. A cohort study^9^ from the USA, again involving over 1000 patients, however, found diabetes to be an independent predictor of complications and death within 30 days of oesophagectomy. The present study supports the findings of the latter study. Higher prevalence of microvascular disease of the kidneys and heart, and poor wound healing may all be important contributors to the development of postoperative complications in diabetic patients. The Dutch cohort study^11^ reported that a history of stroke increased the risk of 90-day mortality after oesophagectomy. This finding is also supported by the present results regarding cerebral disease, although this association must be interpreted cautiously owing to limited statistical power. The lack of association between other malignancy and poor postoperative outcome within 30 days of surgery has been found previously^10^, as well as in the present study of 90-day postoperative outcome. This could reflect conservative selection for surgery in patients with a history of another malignancy.

Despite these limitations, it remains clear that careful assessment to look for co-morbidities, pretreatment optimization and tailored postoperative care are elements that still require investigation in order to improve outcomes for patients needing oesophagectomy.

## Funding

Swedish Cancer Society (grant number 14 0322)

Swedish Research Council (grant number C0324101)

China Scholarship Council (grant number 201808110159)

## Acknowledgements

This study was supported by Swedish Cancer Society, Swedish Research Council, and China Scholarship Council. These study sponsors had no role in the design of the study; the collection, analysis, or interpretation of the data; the writing of the manuscript; or the decision to submit the manuscript for publication.

*Disclosure.* The authors declare no conflict of interest.

## Notes

Presented to the Advances in Health Care Sciences Conference, Stockholm, Sweden, November 2019, and the 27th Annual Conference of the International Society for Quality of Life Research, virtual conference, October 2020
